# Biomechanical Analysis of the Forces Exerted during Different Occlusion Conditions following Bilateral Sagittal Split Osteotomy Treatment for Mandibular Deficiency

**DOI:** 10.1155/2019/4989013

**Published:** 2019-06-02

**Authors:** Yuan-Han Chang, Man-Yee Chan, Jui-Ting Hsu, Han-Yu Hsiao, Kuo-Chih Su

**Affiliations:** ^1^Department of Stomatology, Taichung Veterans General Hospital, Taichung, Taiwan; ^2^School of Dentistry, College of Oral Medicine, Chung Shan Medical University, Taichung, Taiwan; ^3^School of Dentistry, College of Medicine, China Medical University, Taichung, Taiwan; ^4^Department of Biomedical Engineering, Hung Kuang University, Taichung, Taiwan; ^5^Department of Medical Research, Taichung Veterans General Hospital, Taichung, Taiwan

## Abstract

The bilateral sagittal split osteotomy (BSSO) technique is commonly used to correct mandibular deficiency. If the patient is exposed to excessive external forces after the procedure, occlusal changes or nonunion may occur. However, previous studies only focused on single external forces on the mandible and did not conduct relevant research on the forces exerted by different occlusion conditions. The main purpose of this study was to use finite element analysis methods to determine the biomechanics of four common occlusion conditions after BSSO surgical treatment. This study constructed a finite element analysis computer model of a miniplate implanted in the lower jaw. The structure of the model consisted of the mandible, miniplate, and screws. In addition, external forces were applied to the superficial masseter, deep masseter, medial pterygoid, anterior temporalis, middle temporalis, and posterior temporalis muscles to simulate the incisal clench, intercuspal position (ICP), right unilateral molar clench (RMOL), and right group function occlusion conditions. Subsequently, this study observed the effects of these conditions on the miniplate, screws, and mandible, including the von Mises stress values. The results showed that all of the different occlusion conditions that this study evaluated placed high stress on the miniplate. In the ICP and RMOL occlusion conditions, the overall mandibular structure experienced very high stress. The screw on the proximal segment near the bone gap experienced high stress, as did the screw on the buccal side. According to the present analysis, although the data were not directly obtained from clinical practice, the finite element analysis could evaluate the trend of results under different external forces. The result of this study recommended that patients without intermaxillary fixation avoid the ICP and RMOL occlusion conditions. It can be used as a pilot study in the future for providing clinicians more information on the biomechanics of implantation.

## 1. Introduction

In oral surgery clinics, malocclusion and associated facial bone deformities are common and primarily affect the appearance and occlusion of patients. According to previous studies, mandibular deficiency is a common phenomenon, particularly in developing adolescents, with a worldwide prevalence of 1.1-21.5% [1]. To correct mandibular deficiency, mandibular advancement is often performed via intraoral vertical ramus osteotomy (IVRO) and bilateral sagittal split osteotomy (BSSO) [2]. In IVRO, a vertical incision is made in the ramus, which divides the mandible into two parts (anterior and posterior), thereby moving the mandible to achieve a reduction effect. The advantage of IVRO is that the operation is simple and fast. However, a disadvantage is that internal fixation of the mandible cannot be achieved with artificial bone screws, and thus, bone healing must be assisted by intermaxillary fixation. Therefore, patients cannot open their mouths for about 6 to 8 weeks after surgery [3]. As for BSSO, the horizontal cutting line is located above the medial side of the ramus and above the lingua, while the vertical cutting line is located at the distal side of the second molar on the lateral side of the mandible. Next, the two cutting lines are connected along the external oblique ridge. After cutting, the mandible is divided into two parts, a proximal segment and a distal segment, and fixed with miniplates. One advantage of BSSO over IVRO is that the bone has a larger contact area and higher stability. In addition, both mandibular advancement and setback can be achieved with BSSO surgery, as desired. Therefore, mandibular advancement with BSSO is the more common approach for treating mandibular deficiency [4]. Clinically, intermaxillary fixation for several days to weeks after mandibular orthognathic surgery can be performed. However, some physicians prefer to maintain an open airway for the patient after surgery and thus decide against intermaxillary fixation [5]. Consequently, if the mandible without intermaxillary fixation experiences excessive external forces, then changes in occlusion or poor bone healing may occur.

Previously, researchers have used biomechanical methods to evaluate the postoperative efficacy of BSSO. For instance, Hadi et al. [6] performed a general biomechanical analysis of bicortical screws in the mandible. Although their study did not investigate the effects after BSSO surgery, their research methods can be used as a reference framework for performing biomechanical experiments following BSSO surgery. Additionally, Nieblerová et al. [7] and Olivera et al. [8] used minipig and sheep mandibles to investigate the biomechanics of different BSSO reductions. However, the study samples were primarily animal based, and thus, the results of the study may not accurately reflect the situation in the human body. Oguz et al. [9] used a unilateral artificial pseudobone mandibular model to investigate the biomechanical effects of different plate reset patterns through biomechanical methods. Ribeiro-Junior et al. [10] used a similar approach to investigate the effects of different BSSO techniques, revealing that the locking miniplate approach had relatively better stability. Although locking miniplates have a good fixation effect, the prominent plate profile will not be accepted by patients. Therefore, facial bone fixation is still primarily based on miniplates. It should be noted that these studies mainly used in vitro biomechanics to investigate the effects of BSSO surgery, and as such, the external forces applied can only be simulated by a simple, single external force and the effects of muscles on actual chewing cannot be considered. Therefore, it is difficult for the results of in vitro studies to reflect the actual situation of different occlusion movements in human.

With advances in computer technology, finite element analysis has become a commonly used analytical method in the field of dental biomechanics because it can be used to simulate the biomechanics of different structures, materials, and force patterns. A prior study used finite element analysis to investigate the efficacy of different reconstruction methods for the treatment of mandibular defects [11]. Additionally, other researchers have used finite element analysis to investigate various BSSO fixation methods and the effects of different materials (absorbable materials) on the strength and mechanics of fixation [12]. For instance, Erkmen et al. [13] investigated the effects of using miniplates and different fixation methods for advancement surgery, while another study evaluated whether locking miniplates have sufficient strength to complete internal fixation of the mandible [14]. Although many previous studies have used finite element analysis to investigate the biomechanical effects of BSSO fixation and provide recommendations to clinicians, most of the simulated models have been unilateral, the overall mandibular models are incomplete, and the applied external forces are simple, single external forces; hence, the simulated results likely do not reflect the actual conditions. Further, finite element analysis has been employed to investigate the effects of different external forces on implanted artificial total temporomandibular joints, focusing on the incisal clench (INC), intercuspal position (ICP), right unilateral molar clench (RMOL), left unilateral molar clench, right group function (RGF), and left group function occlusion conditions [15]. Therefore, the loading conditions and boundary conditions outlined in the present study will provide a reference for researchers in different occlusion conditions.

As mentioned above, prior studies have demonstrated that BSSO surgery is commonly used to treat mandibular deficiency. However, because most previous in vitro studies used a single external force, the effects of these forces on miniplates under different occlusion conditions cannot be easily measured, and thus, they remain unclear. Hence, the main purpose of this study was to use finite element analysis methods to simulate external forces from the mandibular muscles and investigate the effects of these forces on miniplate implantation under four common occlusion conditions. The results of this study will provide clinicians with mechanical references for different occlusion forces in the overall mandibular structure and miniplate after miniplate implantation in BSSO surgeries, ultimately helping clinicians to avoid surgical failure due to different occlusion conditions.

## 2. Materials and Methods

### 2.1. Building a Simulation Geometry Model

**Figure 1:**
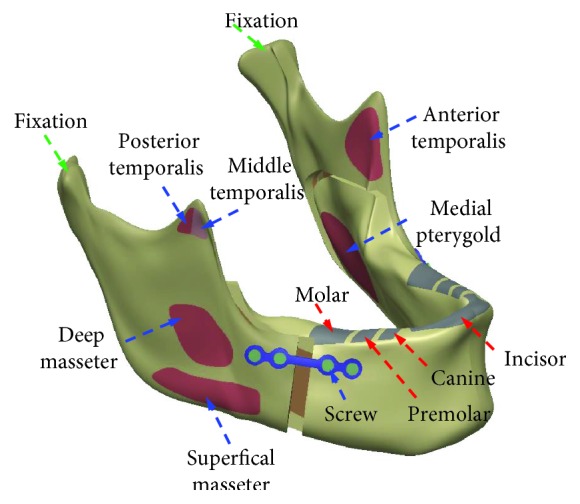
Finite element analysis models and sites experiencing muscular forces in the present study.

**Figure 2:**
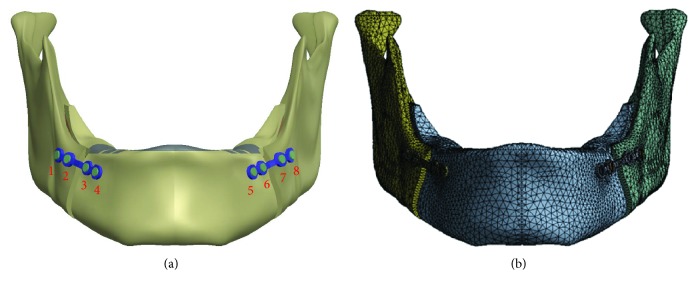
(a) Sites and numbers of the different screws after mandibular miniplate implantation. (b) Mesh of the computer model used in the present study.

This study was designed to investigate the effects of four different occlusion conditions on the miniplate. To do this, this study constructed a finite element analysis computer model of the mandible with the miniplate. The models used in this study included four components, namely, the cortical bone of the mandible, cancellous bone, miniplate, and screws (Figure 1). The appearance of this study-constructed model was based on models of the mandible in previous studies [15], which were primarily composed of cortical bone and cancellous bone. The mandible model established in this study imports the CT images of the mandible to Mimics (Mimics Medical 19.0, Materialise, Leuven, Belgium), selects the peripheral contour of the mandibular cortical bone in Mimics using circles, and retracts the whole model by 2 mm to establish the contour of the cancellous bone structure. This study employed three-dimensional computer-aided design software (SOLIDWORKS 2016; Dassault Systemes SOLIDWORKS Corp., Waltham, MA, USA) for creating and modifying the computer model. The peripheral contours of mandibular cortical and cancellous bones are imported to SOLIDWORKS, and the complete solid model of mandibular cortical bone and cancellous bone is established using the loft function of SOLIDWORKS software. The computer model of the mandible was cut as described in previous studies [10], to simulate the treatment of mandibular deficiency by BSSO surgery. The distal segment was moved outward by 4 mm. In addition, the computer-aided design software SOLIDWORKS was used to combine two miniplates and eight screws (the sites and numbering of the eight different screws are shown in Figure 2(a)). After the three-dimensional computer model was constructed, it was imported into the finite element analysis software (ANSYS Workbench 19.0; ANSYS Inc., Canonsburg, PA, USA) for analysis.

### 2.2. Loading Conditions and Boundary Conditions

This study investigated four different occlusal conditions commonly found in clinical practice, namely, INC, ICP, RMOL, and RGF. Of these conditions, INC primarily simulated contact of the incisal edges, ICP simulated maximum intercuspation of the posterior teeth, RMOL simulated contact of the right (unilateral) posterior teeth, and RGF simulated lateral movement of the right posterior dentition. In finite element analysis, different boundary conditions and loading conditions must be provided based on these four different occlusion conditions. This study based the external force data and application methods on those used in previous studies [15, 16]. For the boundary conditions, the condyle was set as a fixed node and the displacement setting method was used to fix the *x*-, *y*-, and *z*-axis displacements to 0, which allows this point to rotate freely. For the loading conditions, external forces were applied to the superficial masseter (SM), deep masseter (DM), medial pterygoid (MP), anterior temporalis (AT), middle temporalis (MT), and posterior temporalis (PT) muscles (Figure 1). The magnitude and direction of the external forces are shown in Table 1. In addition, fixation was applied at the incisor, canine, premolar, and molar tooth positions in the different occlusion conditions, as shown in Table 1. The sites of contact between the miniplate and screws and between the miniplate and mandible were set to “no separation,” primarily to simulate the lack of separation between these surfaces, but also to allow for slight, frictionless sliding.

### 2.3. Material Properties of the Model

The research model was composed of four parts, namely, the cortical bone, cancellous bone, miniplate, and screws. The material properties used in this study were obtained from previous studies [17].  Table 2 shows the material properties in this study simulation. All materials were assumed to be homogeneous, isotropic, and linear elastic. Two independent parameters, i.e., Young's modulus (*E*) and Poisson's ratio (*ν*), were used to characterize the properties of the materials. The simulated miniplate material was composed primarily of titanium alloy. The bones were divided into the cortical bone and cancellous bone. Moreover, all of the finite element analysis computer models in this study used tetrahedral meshes (Figure 2(b)). The software used for mesh in this study was ANSYS Workbench 19.0. After the meshes passed the convergence test, all models reached the 5% stop criteria of the convergence test [18, 19]. The numbers of nodes and meshes were 144,969 and 74,878, respectively. Therefore, the finite element mesh model used herein to investigate the effects of different occlusion conditions on miniplate implantation was reasonable.

Following finite element analysis, this study utilized the von Mises stress values as an observational index. The von Mises stress distributions of the miniplate, eight screws, and mandibular screw positions on the left and right sides of the mandible were observed to investigate the biomechanical effects of the four different occlusion conditions after BSSO treatment for mandibular deficiency.

## 3. Results

**Figure 3:**
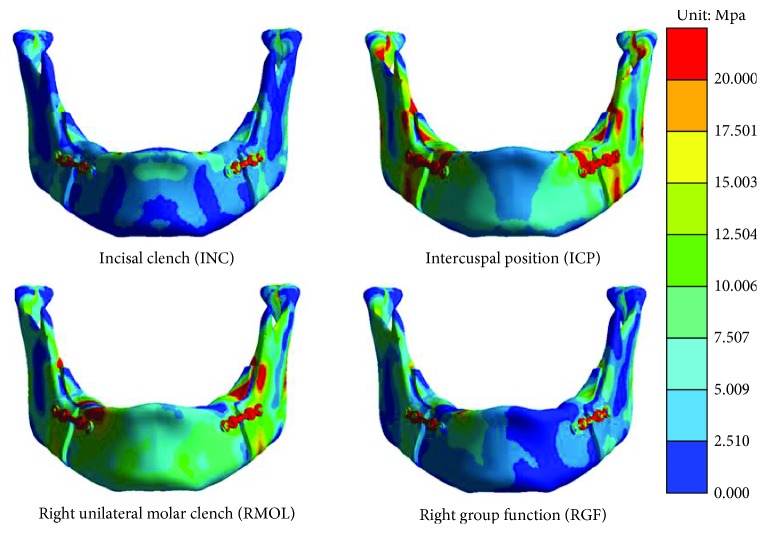
Overall stress distributions in the mandibular region under the four different occlusion conditions.

After finite element analysis, this study obtained the overall stress distributions on the mandible under the four different occlusion conditions, as shown in Figure 3. This figure reveals that high stress occurred at the miniplate under all four occlusion conditions. The mandibular stress was particularly high in the ICP and RMOL conditions.

**Figure 4:**
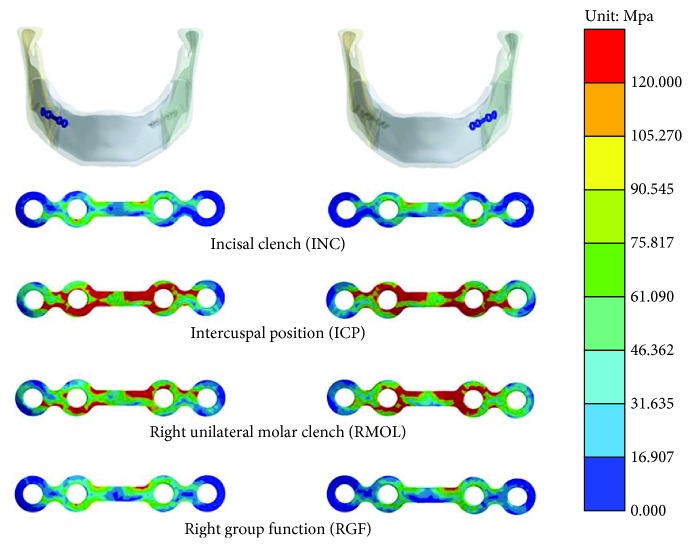
Stress distributions on the right and left miniplates under the four different occlusion conditions.

Figure 4 shows the stress distributions on the left and right miniplates under the four occlusion conditions. High stress primarily occurred in the region between the miniplates (the bone gap at the mandibular joint), especially at the site of the screw in the proximal segment and at the corner of the miniplate in the distal segment. Among the four conditions, the ICP and RMOL conditions in particular put high stress on the miniplate.  Table 3 shows the maximum von Mises stress values for the miniplates in the four different occlusion conditions.

**Figure 5:**
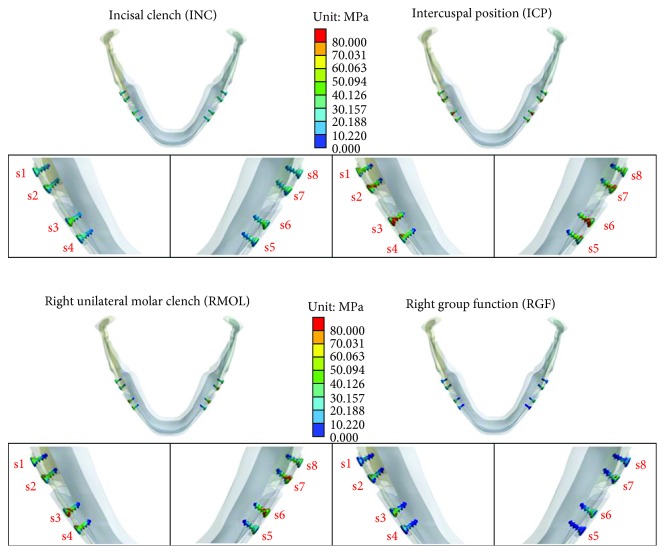
Stress distributions on the eight screws under the four different occlusion conditions.

Figure 5 shows the stress distributions on the eight screws used for miniplate fixation (screw positions numbered as shown in Figure 2(a)). High stress was mainly observed at the junction of the miniplate and screws. Among the four occlusion conditions, higher stress on the screws was observed under the ICP and RMOL conditions, especially near the junction of the screws and miniplate.  Table 4 shows the maximum von Mises stress values on the eight screws under the four different occlusion conditions.

**Figure 6:**
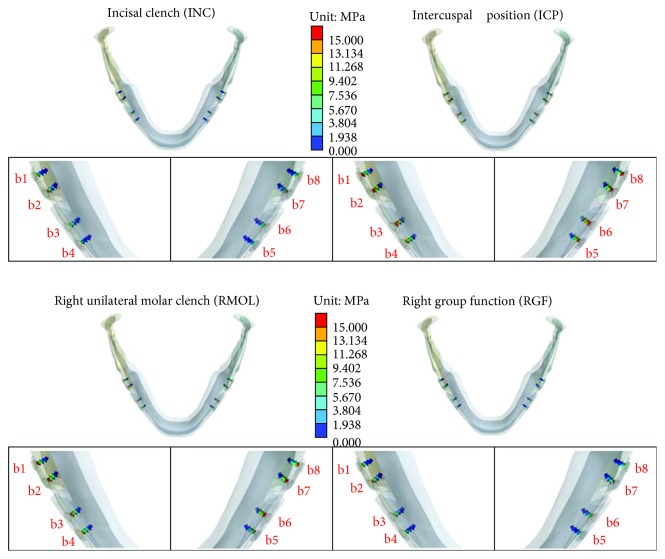
Stress distributions at the screw insertion sites in the mandible under the four different occlusion conditions.

Figure 6 shows the stress distributions on the mandible at the screw insertion positions (b1–b8 indicate the positions of the different screws and the corresponding sites in the mandible). High stress on the mandible was produced primarily on the buccal side. The ICP and RMOL conditions appeared to place higher stress on the mandible than did the other conditions.  Table 5 shows the maximum von Mises stress values on the mandible at the eight screw insertion sites in the four different occlusion conditions.

## 4. Discussion

To treat mandibular deficiencies, the BSSO surgical procedure is often performed. However, this surgical treatment requires destruction of the mandible border, because the mandible border is the strongest part of the mandible and can resist bending forces. Therefore, it is essential that the fixation strength of the miniplate in the mandible is sufficient. Because of the limitations of previous in vitro experiments, including the use of only single forces, conducting relevant research on the effects of different occlusion conditions has been difficult, and thus, no relevant references exist. To help resolve this, the present study used finite element analysis to investigate the strength of the combination of the mandible and miniplate under four common occlusion conditions. The data in the study will provide clinicians with a reference basis for the stress incurred on the screws, miniplate, and mandible under different occlusion conditions following miniplate implantation.

Herein, regarding the overall stress distributions of the mandible, this study noted that following miniplate fixation, the mandible experienced external forces when clenched, which placed high stress on the miniplate. This high stress can primarily be explained by Hooke's law. If the mandible and miniplate were displaced, the stress would be proportional to Young's modulus. In the present study, Young's modulus of the miniplate and cortical bone was 110,000 and 17,000 MPa, respectively, indicating that in the overall structure, the miniplate experienced higher stress. The results of this study show that the values for the miniplate in the four groups are smaller than the yield strength of the titanium alloy (tensile strength of 1100 MPa [20]), and hence, the miniplate is not easily deformed under normal occlusion loading conditions. Further, of the four different occlusion conditions, the INC and RGF conditions placed less stress on the mandible than did the other conditions. The INC condition mainly simulated contact of the incisal edges, and because the muscles that produce this action apply little force, the mandible only experienced some high stress at the incisors. As the RGF condition simulated lateral movement of the right posterior dentition, the vertical force applied by the muscle to the mandible was also small (the force observed on the tooth is about 100 N according to previous literature [16]) and only the right posterior tooth area experienced relatively high stress. In addition, much higher stress is produced on mandible when ICP and RMOL condition. The ICP condition simulated maximum intercuspation of the posterior teeth. According to the previous literature, because of the small lever-arm relationship in the posterior tooth area (with the temporomandibular joint as the fulcrum), a high occlusion force occurs in the posterior tooth area (~700 N) [16], and thus, this study used a large external force for the muscle boundary condition setting. Consequently, obvious high stress was observed in the posterior tooth area of the mandible, along with high stress distributions in the mandible; these results are similar to those reported in previous studies [16]. The RMOL condition simulated contact of the right (unilateral) posterior teeth. This action is an unbalanced occlusion condition. As such, in addition to the occlusal forces concentrated in the posterior tooth area, the external force applied by the muscles is also large. Therefore, high stress occurred at the posterior tooth contact area and throughout the entire mandible. Additionally, since the occlusion is unbalanced, high non-occlusal stress occurred in the left side of the retromolar area, which may have been caused by the high stress that is produced by bending or torsion. After BSSO surgery, the teeth are usually fixed in the ideal occlusion position (intermaxillary fixation); however, to improve patient life quality of post operation and maintain an open airway, many clinicians have opted not to perform intermaxillary fixation in recent years [5]. According to these study findings, after BSSO, patients should be advised to consume liquids and soft foods and to avoid ICP and RMOL occlusion to reduce the force on the mandible.

**Figure 7:**
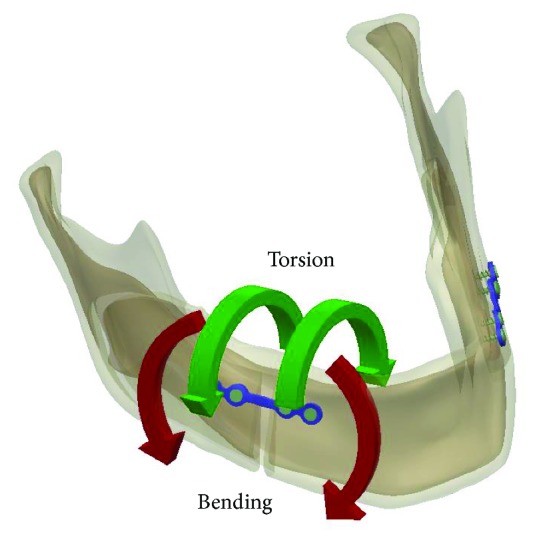
The miniplate region produces two bending forces and two torsion forces.

In the current study, the stress distributions on the miniplate showed that high stress mainly occurred in the middle part of the miniplate (the point of contact with the mandible). As previously indicated by Chuong et al. [21], the primary reason for this is that the miniplate area produces two bending forces and two torsion forces (Figure 7). Since both sides of the miniplate are fixed using screws, the miniplate is affected by torsion in the middle area, thereby producing higher stress. The miniplate is also subjected to an external force. Because of the geometric shape of the miniplate, high stress is generated where the cross-sectional area changes. However, the high stress that this study observed on the miniplate in the present study was less than the yield strength of the titanium alloy, and hence, the miniplate was not deformed under the four different occlusion conditions that this study evaluated.

When observing the stress distributions on the screws, this study found that the two screws near the middle of the miniplate experienced higher stress than did the other screws, likely because the miniplate was being pulled to the screw that provided fixation of the mandible under the external bending and torsion forces. Thus, the two screws close to the middle of the miniplate experienced higher stress, especially the screw which is near the bone gap to the proximal segment (number 2 or 7). These findings suggest that clinicians should pay careful attention to the high stress that may be placed on the screw during miniplate fixation. Further, to achieve overall postimplant stability, a thicker screw should be used or the strength of the miniplate at that site should be enhanced.

When observing the stress distributions in the mandible at the screw insertion sites, this study noticed that the high stress sites were similar to those on the screws, primarily near the buccal side. The principles underlying this high stress are related to Hooke's law. During occlusion, when pulling by the miniplate produces displacement, the screw also pulls on the mandible and the surface of the mandibular screw hole (buccal side) will be deformed. Therefore, according to Hooke's law (the mandible has the same Young's modulus), high stress is produced near the buccal side. The deformation of the screw hole loosens the screw and miniplate, resulting in excessive movement between the bones, causing bone nonunion and potentially surgical failure, which may even require removal of the miniplate and refixation. These study findings indicate that it may be advisable to use thicker screws for fixation to reduce the stress on the screw and the likelihood of mandible deformation (the hole for screw insertion), as this will help to reduce surgical failure and increase the overall stability of the mandible.

This study using finite element analysis to investigate the forces involved when implanting a miniplate to treat mandibular deficiency has some limitations related to the biomechanical analysis of the different occlusion conditions. First, all of the material properties in this study were assumed to be homogeneous, isotropic, and linearly elastic in order to simplify the simulation, and thus, the material properties were set by referring to previous studies [15, 19]. Second, some of the models were simplified in the present study, that is, this study only observed the effects in the mandible, not in the entire skull, and no teeth were constructed. This study opted to use this simplified model because the main area of interest in the current study was the miniplate implantation site and such simplification can reduce the computer simulation time. To perform the stress and strain analysis of the tooth root or periodontal ligament and alveolar bone, we must establish a complete tooth model. However, this study mainly is aimed at investigating the efficacy of miniplate fixation on the mandible after BSSO surgery in 4 common occlusal conditions. This study was not aimed at investigating the biomechanical behavior of the teeth or periodontal areas. Therefore, this study did not consider the establishment of a tooth model. This study only fixed the mandible in the occlusal planes of different tooth positions. The literature has several reports on this method [15, 22]. The model used in this study was mainly designed with reference to previous studies and simulated 4 occlusal modes, namely, INC, ICP, RMOL, and RGF. In this method, we fixed the tooth positions, utilized the external force on the mandible (giving boundary conditions by simulating muscle contractions) and applied restraint (using tooth position fixation as load conditions). However, due to these simplifications, this study finding may not directly replicate reality; nevertheless, clear trends can be identified for the topics of interest in the study.

Here, this study used finite element analysis and visual observations to investigate the biomechanical forces exerted on the miniplate, screws, and mandible under different occlusion conditions. The results showed that among the four evaluated occlusion conditions, the ICP and RMOL conditions in particular stressed and deformed the mandible and miniplate. Although the values identified in the present study are likely different from those in actual clinical situations, these study findings will provide a reference basis both for the maxillofacial surgeons when performing the BSSO surgery to treat mandibular deficiencies and for the patients during the recovery period. Patients should be advised to avoid specific occlusion movements to reduce the incidence of failure after surgical implantation and to improve their prognosis. This study data will also provide a biomechanical basis for the design and development of miniplates in the future.

## 5. Conclusion

The effects of different occlusal conditions on the miniplate, screws, and mandible after BSSO surgery for treating mandibular deficiency were investigated through finite element analysis. The results showed that the implanted miniplate exhibited high stress under various occlusion conditions. In the ICP and RMOL occlusion conditions, the overall mandibular structure experienced very high stress. According to this study model, the screw near the bone gap in the proximal segment experienced high stress. High stress was also generated at the site near the buccal side. The results of the present study, in addition to providing a biomechanical basis for the forces generated on the miniplate, screws, and mandible following BSSO surgery, will also improve the design of miniplates and screws in the future to reduce the stress that is exerted on the screws and mandible. Moreover, patients are advised to avoid ICP and RMOL occlusion in order to reduce the incidence of miniplate implantation failure and to improve patients' prognoses.

## Figures and Tables

**Table 1:** The loading conditions, size, and direction of the muscular forces produced by the different occlusion conditions and sites of tooth fixation in the present study. This table is reproduced from Huang et al. [15] and Korioth and Hannam [16].

Clenching tasks	Side	Direction	Muscular force (N)						Constraint
			SM	DM	MP	AT	MT	PT	
Incisal clench (INC)	Right	Force	76.2	21.2	136.3	12.6	5.7	3.0	Constrained the incisor regions
		Fx	-15.8	-11.6	66.3	-1.9	-1.3	-0.6	
		Fy	-31.9	7.6	-50.9	-0.6	2.9	2.6	
		Fz	67.3	16.1	107.8	12.5	4.8	1.4	
	Left	Force	76.2	21.2	136.3	12.6	5.7	3.0	
		Fx	15.8	11.6	-66.3	1.9	1.3	0.6	
		Fy	-31.9	7.6	-50.9	-0.6	2.9	2.6	
		Fz	67.3	16.1	107.8	12.5	4.8	1.4	

Intercuspal position (ICP)	Right	Force	190.4	81.6	132.8	154.8	91.8	71.1	Constrained the canine and premolar regions
		Fx	-39.4	-44.6	64.6	-23.1	-20.4	-14.8	
		Fy	-79.8	29.2	-49.6	-6.8	45.9	60.8	
		Fz	168.3	61.9	105.1	153.0	76.8	33.7	
	Left	Force	190.4	81.6	132.8	154.8	91.8	71.1	
		Fx	39.4	44.6	-64.6	23.1	20.4	14.8	
		Fy	-79.8	29.2	-49.6	-6.8	45.9	60.8	
		Fz	168.3	61.9	105.1	153.0	76.8	33.7	

Right unilateral molar clench (RMOL)	Right	Force	137.1	58.8	146.8	115.3	63.1	44.6	Constrained the right molars
		Fx	-28.4	-32.1	71.4	-17.2	-14.0	-9.3	
		Fy	-57.4	21.0	-54.8	-5.1	31.5	38.1	
		Fz	121.2	44.5	116.1	114.0	52.8	21.1	
	Left	Force	114.2	49.0	104.9	91.6	64.1	29.5	
		Fx	23.6	26.7	-51.0	13.7	14.2	6.1	
		Fy	-47.9	17.5	-39.1	-4.0	32.0	25.2	
		Fz	101.0	37.1	83.0	90.5	53.6	14.0	

Right group function (RGF)	Right	Force	34.3	29.4	12.2	104.3	61.2	46.9	Constrained the right canine, premolars, and molars
		Fx	-7.1	-16.0	6.0	-15.5	-13.6	9.8	
		Fy	-14.4	10.5	-4.6	-4.6	30.6	40.1	
		Fz	30.3	22.3	9.7	103.0	51.2	22.2	
	Left	Force	51.4	21.2	132.8	11.1	5.7	4.5	
		Fx	10.6	11.6	-64.6	1.7	1.3	0.9	
		Fy	-21.5	7.6	-49.6	-0.5	2.9	3.9	
		Fz	45.4	16.1	105.1	10.9	4.8	2.2	

SM: superficial masseter; DM: deep masseter; MP: medial pterygoid; AT: anterior temporalis; MT: middle temporalis; PT: posterior temporalis. All raw data were obtained from Huang et al. [15] and Korioth and Hannam [16].

**Table 2:** Material properties used in the present study.

	Young's modulus (MPa)	Poisson's ratio
Cancellous bone	1000	0.3
Cortical bone	17000	0.3
Miniplate	110000	0.3
Screw	118000	0.3

**Table 3:** Maximum von Mises stress values on the miniplates.

	Peak von Mises stress values on the right-side miniplates (MPa)	Peak von Mises stress values on the left-side miniplates (MPa)
INC	134.02	153.56
ICP	443.75	491.00
RMOL	302.60	372.80
RGF	157.10	138.58

**Table 4:** Maximum von Mises stress values on the eight screws.

	Peak von Mises stress on the s1 screw (MPa)	Peak von Mises stress on the s2 screw (MPa)	Peak von Mises stress on the s3 screw (MPa)	Peak von Mises stress on the s4 screw (MPa)	Peak von Mises stress on the s5 screw (MPa)	Peak von Mises stress on the s6 screw (MPa)	Peak von Mises stress on the s7 screw (MPa)	Peak von Mises stress on the s8 screw (MPa)
INC	118.950	169.530	120.050	64.761	66.414	116.790	165.190	117.220
ICP	365.220	588.470	406.780	187.420	207.540	392.390	577.830	356.560
RMOL	280.500	356.330	275.180	168.350	177.800	428.110	544.040	230.680
RGF	97.732	222.130	167.530	53.090	67.689	153.370	199.390	67.678

**Table 5:** Maximum von Mises stress values at the screw insertion sites.

	Peak von Mises stress at b1 on the mandible (MPa)	Peak von Mises stress at b2 on the mandible (MPa)	Peak von Mises stress at b3 on the mandible (MPa)	Peak von Mises stress at b4 on the mandible (MPa)	Peak von Mises stress at b5 on the mandible (MPa)	Peak von Mises stress at b6 on the mandible (MPa)	Peak von Mises stress at b7 on the mandible (MPa)	Peak von Mises stress at b8 on the mandible (MPa)
INC	20.324	39.124	29.273	15.191	26.173	25.474	39.034	20.462
ICP	66.423	126.500	102.480	43.784	52.865	86.761	125.960	66.597
RMOL	48.194	82.709	61.630	48.399	115.490	98.718	109.400	65.414
RGF	22.321	41.563	39.653	14.452	30.620	26.072	38.142	16.680

## Data Availability

The data used to support the findings of this study are available from the corresponding author upon request.
